# Outbreak of Hepatitis A Linked to Frozen Blueberries, the Netherlands, 2024–2025

**DOI:** 10.3201/eid3208.251406

**Published:** 2026-08

**Authors:** Ingrid H.M. Friesema, Ingeborg L.A. Boxman, Ife A. Slegers–Fitz-James, Heather E. van Brug, Jessica C.J. Elzakkers, Gregorius J. Sips, Ellen P.A. Verspui-van der Eijk, Dana L.L. Adriaansens, Florien Dusseldorp, Sabiena G. Feenstra, Harry Vennema, Eelco Franz

**Affiliations:** National Institute for Public Health and the Environment, Bilthoven, the Netherlands (I.H.M. Friesema, E.P.A. Verspui-van der Eijk, D.L.L. Adriaansens, F. Dusseldorp, S.G. Feenstra, H. Vennema, E. Franz); Wageningen Food Safety Research, Wageningen University and Research, Wageningen, the Netherlands (I.L.A. Boxman); Netherlands Food and Consumer Product Safety Authority, Utrecht, the Netherlands (I.A. Slegers–Fitz-James); Public Health Service Rotterdam-Rijnmond, Rotterdam, the Netherlands (H.E. van Brug, J.C.J. Elzakkers, G.J. Sips); Public Health Service Zuid-Holland Zuid, Dordrecht, the Netherlands (E.P.A. Verspui-van der Eijk)

**Keywords:** hepatitis A, viruses, food safety, outbreak, blueberries, the Netherlands

## Abstract

Twenty-four patients in the Netherlands who became ill during November 2024–February 2025 were part of a hepatitis A virus genotype IA cluster. Consumption data combined with detection of RNA of the hepatitis A outbreak strain in a food product pointed toward frozen blueberries from a specific supermarket chain as the source.

Hepatitis A is an acute, self-limiting liver disease transmitted primarily via the fecal–oral route. Risk factors for infection include consumption of contaminated food or water and person-to-person contact. In Western Europe, hepatitis A endemicity is low (*1*) and is associated primarily with travel to endemic countries (*2*) or consumption of contaminated, imported food (*3*).

In foodborne outbreaks, investigators often use questionnaires to link patients based on consumption of a specific food product and employ genotyping to identify genetically related cases. On December 31, 2024, public health officials in the Netherlands identified a third case of hepatitis A with a specific genotype IA strain, referred to as the outbreak strain. One week later, the cluster expanded to 9 cases, and all case-patients reported consumption of frozen fruits bought from 1 specific supermarket chain, triggering an outbreak investigation.

## The Investigation

Hepatitis A is a notifiable disease in the Netherlands. Accordingly, regional Public Health Services must collect relevant patient data and report them, pseudonymized, to the National Institute of Public Health and the Environment (*4*). As part of the molecular surveillance of hepatitis A virus (HAV), the agency also requests laboratories to send HAV-positive serum and stool samples for further testing, including sequencing according to the HAVNET typing protocol (*5*). For the outbreak we report, we defined an outbreak case as a person infected by an IA strain of hepatitis A sharing at least 459/460 nt with the outbreak strain and a disease onset from November 2024 onward. This outbreak investigation was conducted as part of routine public health surveillance and so was exempt from ethical approval. We submitted 2 representative sequences of nearly complete genomes to GenBank (accession nos. PZ095824 and PZ095825). 

In addition to conducting standard interviews with notified outbreak case-patients, 24 in total, we also developed and administered a questionnaire featuring advertisement pictures of all packaged frozen fruits sold by the suspected supermarket chain, following previous methods (*6*). We were unable to collect food consumption data for 1 patient and determined another patient to be likely infected through person-to-person contact with another outbreak case-patient. We determined that the remaining 22 patients had consumed a specific brand of frozen fruit purchased at the same supermarket chain. Nineteen patients reported consuming frozen blueberries, 14 of which identified a specific package. The remaining 3 of the 22 patients reported consuming bilberries, blackberries, or a mix that also contained blueberries, but those patients were not available for further questioning about specific consumption of blueberries.

Food safety inspectors collected leftover packages of blueberries (6 lots, n = 10 packages), raspberries (n = 1), and mixed soft fruits (n = 1) from the homes of 4 outbreak case-patients. Laboratory technicians distributed the contents by weight into multiple subsamples of 25 grams, depending on the availability. We performed analysis for the presence of HAV RNA on 66 subsamples, according to ISO15216–2:2019 under accreditation of the Dutch Council for Accreditation. Before real-time quantitative reverse-transcription PCR (qRT PCR), we treated all RNA samples using the OneStep PCR Inhibitor Removal Kit (Zymo Research, https://www.zymoresearch.com) to reduce inhibitory substances and increase the detectability of the target RNA (*7*). For quantification, we ran a 10-fold dilution of a dsDNA standard (ISO 15216–1:2017) in parallel with some samples. We applied the HAVNET typing protocol in sequencing the obtained HAV RNA (*5*).

We detected HAV RNA in all 10 subsamples from 2 opened packages, containing solely blueberries, received from 1 patient. On the basis of the cycle quantification values, we estimated contamination levels for the two 25-g samples at 3.2 × 10^2^–3.1 × 10^3^ and 1.6 × 10^2^–7.6 × 10^2^ genome copies. We obtained a 446-bp and a 448-bp consensus fragment for the 2 samples, resulting in a 100% match with the outbreak strain over the full lengths of the obtained fragments. We detected no HAV RNA in the remaining 56 subsamples. The packages that tested positive for HAV shared the same expiration date and had time stamps within a short time period. We did not detect HAV RNA in a third bag with the same expiration date but packed later that day.

The epidemiologic evidence and HAV detection in 2 opened bags led the retailer to initiate an extensive recall of the specific batch of frozen blueberries, including batches of the same package size (1 kg) with an earlier expiration date. We shared supplier-related data via the Rapid Alert System for Food and Feed, a European Union mechanism for the rapid exchange of information on food and feed safety risks. The batch of suspected blueberries originated from a single grower. The batch, in its entirety, had been repacked in packages of 1 kilogram for the implicated supermarket chain and distributed to branches in southern regions of the Netherlands.

No reports emerged of patients with onset dates later than 1 incubation period after the recall on January 13, 2025, who also met the outbreak case definition. The outbreak of hepatitis A we report involved 24 patients, 13 male and 11 female, with a median age of 41 years (range 16–77 years). Onset of disease covered a range of November 26, 2024–February 10, 2025 (Figure), and the peak occurred December 11–22 (n = 11). Case-patients resided in areas spanning 9 Public Health Services regions and including 3 contiguous southern provinces in the Netherlands, which overlapped with the geographic distribution of the product. Eight (33%) patients were admitted to a hospital; no deaths were recorded.

**Figure:**
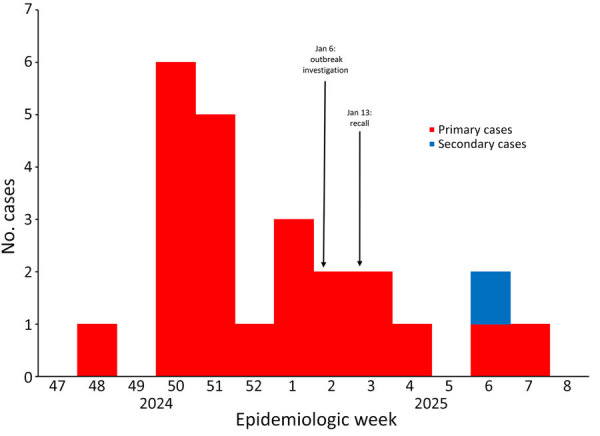
Epicurve of a hepatitis A outbreak linked to frozen blueberries, the Netherlands, 2024–2025. Number of cases per week based on date of disease onset. Cases occurred during November 26, 2024 (epidemiologic week 48), through February 10, 2025 (epidemiologic week 7).

## Conclusions

Berries are a known risk product for foodborne viral diseases (*8*,*9*). Risk factors for contamination include contaminated irrigation or washing water, manual harvesting, and consumption without prior heating. Good personal and environmental hygiene are therefore key to preventing contamination (*8*,*10*). However, sanitary facilities in picking fields and the accommodations of berry pickers might not always meet hygiene standards. We were unable to establish the initial source and route of contamination in the outbreak we investigated.

Detection of HAV RNA in food is complicated by the often heterogenous distribution of the virus within the product (tested 25 g at a time), the levels of contamination often being very low, and characteristics of the food product that can hamper extraction and detection of the viral RNA. Moreover, because of the long incubation period of hepatitis A (up to 7 weeks), leftovers of the implicated batch are often not available for testing. Success factors in our outbreak investigation could have been the relatively large package size (1 kg) and the product being frozen, which both increase the likelihood of having leftover product available for testing. Compared with a smaller package, a large package with low levels of viral contamination might pose a higher risk to the consumer because of repeated exposures over time.

Based on epidemiologic evidence, a specific brand of frozen blueberries was highly likely the source of infection in the outbreak we report. This conclusion was strengthened by microbiologic evidence identifying the same HAV sequences in the berry samples as in the patients. Although we cannot fully rule out the possibility that the positively tested blueberries were contaminated by the patients handling the bags, we find it notable that all 10 subsamples of the 2 contaminated bags tested positive for HAV. Moreover, all case-patients who responded to the additional questionnaire mentioned the specific package, and the food traceback investigation further strengthened the link to the suspected source.

## References

[1] Jacobsen KH. The global prevalence of hepatitis A virus infection and susceptibility: a systematic review. Geneva, Switzerland: World Health Organization; 2009 [cited 2025 Oct 6] https://iris.who.int/server/api/core/bitstreams/25cefdfa-c43a-4315-b86d-7766a372f91f/content

[2] Beauté J, Westrell T, Schmid D, Müller L, Epstein J, Kontio M, et al. Travel-associated hepatitis A in Europe, 2009 to 2015. Euro Surveill. 2018;23:1700583. 10.2807/1560-7917.ES.2018.23.22.1700583 29871720 PMC6152172

[3] Gossner CM, Severi E. Three simultaneous, food-borne, multi-country outbreaks of hepatitis A virus infection reported in EPIS-FWD in 2013: what does it mean for the European Union? Euro Surveill. 2014;19:20941. 10.2807/1560-7917.ES2014.19.43.20941 25375903

[4] Friesema IHM, Sonder GJ, Petrignani MWF, Meiberg AE, van Rijckevorsel GG, Ruijs WL, et al. Spillover of a hepatitis A outbreak among men who have sex with men (MSM) to the general population, the Netherlands, 2017. Euro Surveill. 2018;23:1800265. 10.2807/1560-7917.ES.2018.23.23.1800265 29897040 PMC6152169

[5] National Institute of Public Health and the Environment. HAVNET Hepatitis A Lab Network [cited 2025 Aug 20]. https://www.rivm.nl/en/Topics/H/HAVNET

[6] Mollers M, Boxman ILA, Vennema H, Slegers-Fitz-James IA, Brandwagt D, Friesema IH, et al. Successful use of advertisement pictures to assist recall in a food-borne hepatitis A outbreak in The Netherlands, 2017. Food Environ Virol. 2018;10:272–7. 10.1007/s12560-018-9347-3 29728977 PMC6096949

[7] Boxman I, Hägele G, Jansen C. Improvement of virus extraction from soft fruit by implementing a PCR inhibitor removal kit. Abstract T4–12. Presented at: International Association for Food Protection; St. Louis, Missouri, USA; 2026 Aug 1 [cited 2025 Aug 20]. https://www.foodprotection.org/annualmeeting/archive/2016

[8] Bozkurt H, Phan-Thien KY, van Ogtrop F, Bell T, McConchie R. Outbreaks, occurrence, and control of norovirus and hepatitis A virus contamination in berries: a review. Crit Rev Food Sci Nutr. 2021;61:116–38. 10.1080/10408398.2020.1719383 32008374

[9] Nasheri N, Vester A, Petronella N. Foodborne viral outbreaks associated with frozen produce. Epidemiol Infect. 2019;147:e291. 10.1017/S0950268819001791 31625499 PMC6813648

[10] Maunula L, Kaupke A, Vasickova P, Söderberg K, Kozyra I, Lazic S, et al. Tracing enteric viruses in the European berry fruit supply chain. Int J Food Microbiol. 2013;167:177–85. 10.1016/j.ijfoodmicro.2013.09.003 24135674

